# Effects of a phytobiotic-based additive on the growth, hepatopancreas health, intestinal microbiota, and *Vibrio parahaemolyticus* resistance of Pacific white shrimp, *Litopenaeus vannamei*

**DOI:** 10.3389/fimmu.2024.1368444

**Published:** 2024-08-09

**Authors:** Qiang Ma, Guiping Zhao, Jiahao Liu, I-Tung Chen, Yuliang Wei, Mengqing Liang, Ping Dai, Waldo G. Nuez-Ortin, Houguo Xu

**Affiliations:** ^1^ State Key Laboratory of Mariculture Biobreeding and Sustainable Goods, Yellow Sea Fisheries Research Institute, Chinese Academy of Fishery Sciences, Qingdao, China; ^2^ Adisseo Life Science (Shanghai) Co., Ltd, Shanghai, China

**Keywords:** shrimp, Immunity, hepatopancreas health, intestinal microbiota, *Vibrio*

## Abstract

*Vibrio* genus is a common pathogen in aquaculture and causes acute hepatopancreatic necrosis disease (AHPND) and massive mortality of shrimp. Many studies have suggested that a single functional ingredient such as plant extract or organic acid can reduce the dependence on antibiotics and promote the growth and immunity of aquatic animals. In this study, we evaluated the effects of a phytobiotic-based compound additive (Sanacore® GM, SNGM), which had a successful trajectory of commercial application in fish farming. However, its effects on the hepatopancreas health and intestinal microbiota of shrimp after *Vibrio* challenge have not been well evaluated. In the present study, Pacific white shrimp were fed diets with or without supplementation of SNGM, and the SNGM grades were 0-g/kg (CON), 3-g/kg (SNGM3), and 5-g/kg (SNGM5) diets. The feed trial lasted 60 days, after which a *Vibrio parahaemolyticus* challenge was performed. The results showed that compared to the CON group, both the SNGM3 and SNGM5 groups had a significantly higher weight gain and a lower feed conversion ratio as well as higher survival after *Vibrio parahaemolyticus* challenge. In the growth trial, the SNGM3 group had a significantly increased total protein, albumin concentration, and acid phosphatase activity in hemolymph compared to the CON group. In the challenge experiment, the SNGM3 and SNGM5 groups had increased albumin and glucose contents as well as the activities of phenoloxidase, lysozyme, alkaline phosphatase, and superoxide dismutase in hemolymph. Both the SNGM3 and SNGM5 groups had improved morphology of the hepatopancreas and intestine. The SNGM5 group had alleviated gut microbiota dysbiosis induced by *Vibrio* infection by increasing the potential probiotic bacterium abundance (*Shewanella*) and decreasing the potential pathogenic bacteria abundance (*Vibrio*, *Photobacteriuma*, *Pseudoalteromonas*, and *Candidatus_Bacilloplasma*). In conclusion, the dietary phytobiotic-based additive at 3-g/kg level increased the growth and *Vibrio parahaemolyticus* resistance of Pacific white shrimp by promoting immune-related enzyme activities and improving the morphological structure of the hepatopancreas and intestine and the intestinal microbiota composition.

## 1 Introduction

Pacific white shrimp (*Litopenaeus vannamei*) is the most popular aquaculture crustacean due to its high nutritional and economic value. With the development of high-density intensive farming, bacterial and viral diseases in shrimp have become a limiting factor for production. *Vibrio* genus is a common pathogen in aquaculture and can result in serious economic loss (1). Currently, acute hepatopancreatic necrosis disease (AHPND), caused by *Vibrio parahaemolyticus*, is a severe epidemic disease in white shrimp aquaculture and results in high mortality (2). Antibiotic has been a main solution for this disease threat. However, frequent and massive use of antibiotics can lead to the emergence of antibiotic-resistant bacteria. In particular, the antibiotic resistance of *Vibrio*, such as ampicillin, ceftriaxone, and imipenem, isolated from farmed shrimp has been widely reported (3). Meanwhile, antibiotics also have some negative effects on animals and threaten human health via their residue in aquatic food products (4). Therefore, searching for strategies which can reduce the dependence on antibiotics has become an urgent task in shrimp farming.

Unlike antibiotics, health additives based on natural components such as plant extracts, organic acids, and yeast extracts have less impact on the antibiotic resistance of microorganisms and are therefore being more and more frequently used in aquaculture (5, 6). The main effective components of plant extracts are polysaccharides, phenolic compounds, flavonoids, alkaloids, terpenoids, caffeic acid, saponins, and essential oils. They are effective for disease control and have also been widely used in human medicine (7–9). In Pacific white shrimp, feeding with diets supplemented with 20 g/kg Asteraceae plant (*Bidens alba*) or Indian borage (*Plectranthus amboinicus*) extract for 7 days significantly increased the total hemocyte count, phenoloxidase activity, superoxide anion production, phagocytic activity, immune gene expression, and resistance to *Vibrio alginolyticus* (10). In black tiger shrimp (*Penaeus monodon*) and freshwater crab (*Paratelphusa hydrodomous*), the injection treatment with five Indian traditional medicinal plant extracts (*Aegle marmelos*, *Cynodon dactylon*, *Lantana camara*, *Momordica charantia*, and *Phyllanthus amarus*, 100 or 150 mg/kg body weight) individually resulted in a higher antiviral activity against white spot syndrome virus (WSSV) (11). Studies have demonstrated that individually adding *Allium sativum*, *Olea europaea*, *Argemone mexicana*, *Sargassum cristaefolium*, *Nigella sativa*, *Phyllanthus amarus*, or *Eleutherine bulbosa* extract in the diet all promoted the immunity and resistance to WSSV or *Vibrio* genus in Pacific white shrimp (12). Organic acids, including formic acid, butyric acid, citric acid, malic acid, fumaric acid, etc., have been commonly used in the food industry and animal feeds. Adding formic acid (0.023%), acetic acid (0.041%), propionic acid (0.03%), and butyric acid (0.066%) could effectively inhibit the growth of *Vibrio harveyi* isolated from sick shrimp on Muller–Hinton agar medium (13). In black tiger shrimp, a novel microencapsulated organic acid blend supplementation in the diet increased the digestibility, survival after *Vibrio harveyi* challenge, and phenol oxidase activity and reduced hepatopancreatic damage, total viable bacteria, and presumptive *Vibrio* spp. counts in the hepatopancreas and intestine (14). Yeast extracts contain nucleotides, amino acids, polysaccharides, and trace elements and have been widely used in animal feeds (15). In kuruma shrimp (*Marsupenaeus japonicus*), the oral administration or injection of a nucleotide-rich baker’s yeast extract increased the gene expressions of anti-microbial peptides/proteins, such as penaeidin, crustin, and lysozyme, and resistance to *Vibrio nigripulchritudo* infection (16). In Pacific white shrimp, low-fish meal diets supplemented with yeast extract (500–1,500 mg/kg) improved the growth, antioxidant enzyme activities, and intestinal morphology (17). In summary, plant extract, organic acid, and yeast extract have been widely used in aquaculture as antibiotic alternative disease control strategies.

Nonetheless, many studies have evidenced that the individual use of plant extract, organic acid, yeast extract, and other immuno-stimulants could increase the disease resistance of shrimp. Effective products, in particular, those based on the combination of functional ingredients, are less studied and applied in the aqua-feed industry. Sanacore® GM (SNGM) is a phytobiotic-based compound additive with a successful trajectory of commercial application in aqua-feeds. Studies on gilthead seabream (*Sparus aurata*) have evidenced that 0.4–0.5% SNGM supplementation increased the resistance to *Enteromyxum leei* and *Vibrio alginolyticus* by improving antioxidation and immunity (18, 19) and also reduced the amount of fishmeal in the feed (20). Meanwhile, dietary 0.2–0.3% SNGM in the Pacific white shrimp also promoted growth performance and resistance to *Fusarium solani* by improving the feed utilization, antioxidant capacity, non-specific immune response, and intestinal health (21). However, few studies have investigated the effects of SNGM on the hepatopancreas health and intestinal microbiota of Pacific white shrimp, in particular, after *Vibrio* challenge. Therefore, the present study aimed to evaluate the efficacy of SNGM in the diets of Pacific white shrimp in terms of growth, immunity, hepatopancreas health, intestinal microbiota, and resistance to *Vibrio parahaemolyticus* challenge. The results of this study could be helpful to the green and healthy development of shrimp farming.

## 2 Materials and methods

### 2.1 Experimental diets

The basal diet (CON) used fishmeal, soybean meal, peanut cake meal, and Antarctic krill meal as main protein sources, and fish oil and soy lecithin as the main lipid sources (**Table 1**). The control diet contained approximately 43% protein and 8.5% lipid. A phytobiotic-based, health-promoting additive compound, Sanacore® GM [SNGM, Adisseo Life Science (Shanghai) Co., Ltd.], which is a mixture of herbal extracts, organic acids, inactivated yeast, and yeast extracts on a mineral carrier, was added into the control diet at the levels of 0.3% and 0.5%, respectively, to obtain the other two experimental diets (designated as SNGM3 and SNGM5, respectively).

**Table 1:** Formulation and proximate composition of the experimental diets (g/kg).

Ingredient	CON	SNGM3	SNGM5
Fish meal	160	160	160
Soybean meal	300	300	300
Peanut cake meal	180	180	180
Antarctic krill meal	50	50	50
Wheat gluten meal	40	40	40
High gluten flour	190	187	185
Fish oil	20	20	20
Soy lecithin	25	25	25
Monocalcium phosphate	14	14	14
Mineral premix^a^	5	5	5
Vitamin premix^a^	10	10	10
L-ascorbyl-2-polyphosphate	2	2	2
Choline chloride	2	2	2
Lysine hydrochloride	1	1	1
Methionine	1	1	1
Sanacore® GM^b^	0	3	5
Total	1,000	1,000	1,000
Proximate composition (% dry matter)			
Crude protein	43.36	43.41	43.74
Crude lipid	8.59	8.87	8.13
Ash	8.22	8.40	8.54

a Vitamin premix and mineral premix, designed for marine shrimp, were purchased from Qingdao Master Biotech Co., Ltd., Qingdao, China.

b Sanacore® GM is a broad-spectrum health-promoting additive consisting of a mixture of herbal extracts, organic acids, inactivated yeast, and yeast extracts on a mineral carrier provided by Adisseo Life Science (Shanghai) Co., Ltd.

The experimental diets were made using a customized single-screw pelleting machine in the laboratory. Before drying, the pellets (2.0 × 2.0 mm) were steamed for 10 min to enhance their stability in water. The diets were then oven-dried to a moisture content of around 5% and were stored at -20°C.

### 2.2 Experimental shrimp and feeding procedure

The juvenile shrimp used in the study were purchased from Rizhao Tengyun Aquaculture Co. Ltd. (Rizhao, Shandong Province, China), where the growth trial was conducted. Flow-through deep-well seawater was used in both the acclimating period and the whole growth trial period. A total of 1,200 experimental shrimp with an average initial body weight of approximately 4.5 g was distributed into 12 polyethylene tanks (300 L). Each diet was randomly assigned to for replicate tanks, and each tank was stocked with 100 shrimp. The growth trial lasted 60 days, and the experimental shrimps were hand-fed to apparent satiation four times each day (6:00, 11:30, 17:00, and 21:30). Dead shrimps were taken out when found, and the number and the weight of dead shrimp were recorded. The residual feed and feces were siphoned out 30 min after every feeding (after the shrimp stopped feeding). During the feeding trial, the water temperature ranged from 24°C to 27°C, salinity was 18 to 20, pH was 8.0 to 8.4, dissolved oxygen was 6 to 7 mg L^-1^, and ammonia was <0.2 mg L^-1^.

### 2.3 Challenge experiment

At the end of the growth trial, 30 shrimps/tank were transferred to separate tanks for the challenge experiment with *Vibrio parahaemolyticus*. A pre-experiment was conducted to determine the most suitable challenging method and corresponding semi-lethal concentration (22). The final challenge method was as follows: shrimps were immersed in 2-L bacteria solution (at a concentration of 1 × 10^8^ cfu/mL) for 30 min. After that, the shrimp-and-bacteria solution was poured into 200 L of clean seawater (the final bacteria concentration was approximately 1×10^6^ cfu/mL). During the challenge experiment, no more clean water was added into the tanks. The mortality was recorded daily continuously for 7 days.

### 2.4 Sample collection

Samplings were conducted for both the growth trial and the challenge experiment. At the end of the growth trial, the weight and the number of shrimps in each tank were recorded after having been anesthetized with eugenol (1:10,000). From each tank, six shrimps were randomly sampled to collect the hemolymph from pericardial cavity using syringes. The hemolymph was centrifuged at 4°C to obtain the supernatant. The hemolymph of three shrimps from each tank was pooled and gently mixed with anticoagulant to count the hemocyte. After that, these nine shrimps were dissected on ice. The hepatopancreas, intestine, and muscle were collected and frozen immediately with liquid nitrogen and then stored at -80°C for further processing. The sampling process after the 7-day challenge experiment was the same as described above.

### 2.5 Proximate composition analysis

The proximate composition analysis of experimental diets, whole fish, and muscle was performed according to the standard methods of the Association of Official Analytical Chemists (2005). Briefly, samples were oven-dried at 105°C to a constant weight for moisture assay. Crude protein content (N × 6.25) was measured by the Kjeldahl method using the UDK142 automatic distillation unit (VELP, Usmate, MB, Italy). The crude lipid content was detected by petroleum ether extraction using the Soxhlet method (Foss Tecator, Hoganas, Sweden). The ash content was measured by incinerating the sample in a muffle furnace at 550°C to constant weight.

### 2.6 Biochemical indexes of the hemolymph, hepatopancreas, and intestine

The contents of total soluble protein (TP, A045–4-2), albumin (ALB, A028–2-1), glucose (GLU, F006–1-1), total cholesterol (T-CHO, A111–1-1), triglycerides (TG, A110–1-1), malonaldehyde (MDA, A003–1-1), and protein carbonyl (PC, A087–1-1) and the activities of alkaline phosphatase (AKP, A059–2-1), acid phosphatase (ACP, A060–2-1), lysozyme (LZM, A050–1-1), phenol oxidase (PO, H247–1-1), and superoxide dismutase (SOD, A001–3-2) in the hemolymph, hepatopancreas or intestine were analyzed by using commercial kits purchased from Nanjing Jiancheng Bioengineering Institute (Nanjing, China). All measurement steps were based on the manufacturer’s protocols (http://www.njjcbio.com/).

### 2.7 Histological analysis of the hepatopancreas and intestine

The middle intestine and hepatopancreas were collected and fixed in 4% paraformaldehyde immediately. Then, the samples were conducted with a graded ethanol series and xylene solution. After paraffin embedding, the molds were sectioned for 5-μm thickness by a semiautomatic microtome. The sections were stained with the Harris hematoxylin-eosin mixture (H&E) and photographed under a light microscope (Nikon Ds-Ri2, Boston, USA).

### 2.8 Intestinal microbiota analysis

The whole intestines of three shrimps from the same tank were pooled as one sample (three tanks were randomly selected from each treatment) under sterile conditions. The total bacterial DNA of the intestine was isolated using marine animals DNA kit (Tiangen, China). The quantity and the quality of extracted DNA were tested by using Titertek-Berthold Colibri spectrometer (Colibri, Germany). The ratio of 260/280 nm of DNA ranged from 1.8 to 1.9 and was confirmed by agarose gel electrophoresis. 338F (5′-GTGCCAGCMGCCGCGGTAA-3′) and 806R (5′-CCGTCAATTCCTTTRAGTTT-3′) were selected as the forward and reverse primer, respectively, and the V3–V4 regions of the bacteria 16S ribosomal RNA genes were amplified by PCR using the DNA as template. The PCR (25 μL volume) was performed by mixing 20 ng DNA, 0.4 μL Fast PfuPolymerase (TransGen Biotech, China), 1 μL of each primer (5 μM), 4 μL 5 × Fast Pfu Buffer, and 2 μL dNTPs (2.5 mM). The PCR program was as follows: 95°C for 5 min, 30 cycles of 95°C for 30 s, 55°C for 30 s, and 72°C for 50 s and 72°C for 10 min. Then, Illumina high-throughput sequencing was carried out by Majorbio Bio-Pharm Technology Co., Ltd. (Shanghai, China). All sequencing data were uploaded to the NCBI database under accession number SUB14101752/PRJNA1055185 and bioinformation cloud platform of Majorbio for further analysis (http://www.i-sanger.com). Cluster analysis was carried out for non-repeating sequences (excluding single sequences) according to 97% similarity using Uparse software, and chimeras were removed in the clustering process to obtain the representative sequences of operational taxonomic units (OTUs). The taxonomic analysis of OTU representative sequences was performed by using RDP classifier. The richness and the diversity of bacteria at each taxonomic level were analyzed by using Qiime and Mothur software.

### 2.9 Statistical analysis

The calculations used in this study include the following:


Weight gain (WG, %)=100×(W1–W0)/W0

Feed conversion ratio (FCR)=feeds consumed/(W1–W0)

Feed intake (FI, %/day)=100×feed intake/[(W1+W0)/2]/days

$$\text{Condition factor }\left(\text{K},\text{ g}/{\text{cm}}^{3}\right)=\text{final body weight}/{\text{final body length}}^{3}$$

Survival (%)=100×final number of shrimp/initial number of shrimps

where W0 = initial body weight and W1 = final body weight.

All data were subjected to one-way analysis of variance (ANOVA) using the SPSS Statistics 20.0 software for Windows. Shapiro–Wilk and Levene’s tests were performed to evaluate the normality and homogeneity of data. Significant differences between the means were detected by using Tukey’s multiple-range test. The results were presented as means ± standard errors of means (SEM), and the level of significance was chosen at *P* < 0.05. In the normal growth experiment, values with different lowercase superscript letters are significantly different (*P* < 0.05). In the challenge experiment, values with different capital superscript letters are significantly different (*P* < 0.05).

## 3 Results

### 3.1 Growth performance and *Vibrio parahaemolyticus* resistance

**Figure 1:**
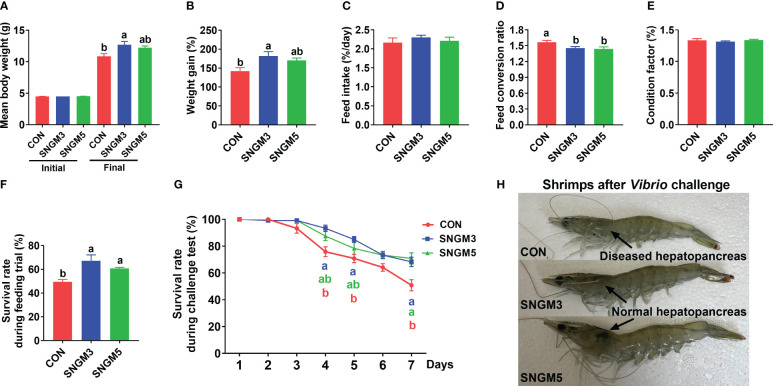
Effects of SNGM on growth performance and *Vibrio parahaemolyticus* resistance. **(A)** Initial and final body weight of the shrimp. **(B)** Weight gain. **(C)** Feed intake. **(D)** Feed conversion ratio. **(E)** Condition factor. **(F)** Survival during growth trial. **(G)** Survival during the 7-day challenge test. **(H)** Color of the hepatopancreas and the appearance of the shrimps after the 7-day challenge test. Values are means ± SEM and different lowercase letters indicate significant difference (*P* < 0.05) between groups.

After the 60-day growth trial, shrimps fed SNGM3 and SNGM5 had significantly higher final body weight (FBW), weight gain (WG), and survival and significantly lower feed conversion ratio (FCR) compared to the CON group (*P* < 0.05) (**Figures 1A, B, D, F**). There were no significant differences in initial body weight, feed intake, and condition factor between the CON and SNGM groups (**Figures 1A, C, E**).

After the 7-day challenge experiment, the survival rates in the SNGM3 and SNGM5 groups were significantly higher than in the CON group (*P* < 0.05) (**Figure 1G**). As shown in **Figure 1H**, the color of the hepatopancreas in the CON group turned white after *Vibrio parahaemolyticus* infection, but the SNGM groups, especially the SNGM5 group, had more normal and healthier (darker color) hepatopancreas after the 7-day challenge experiment.

### 3.2 The body proximate compositions

**Figure 2:**
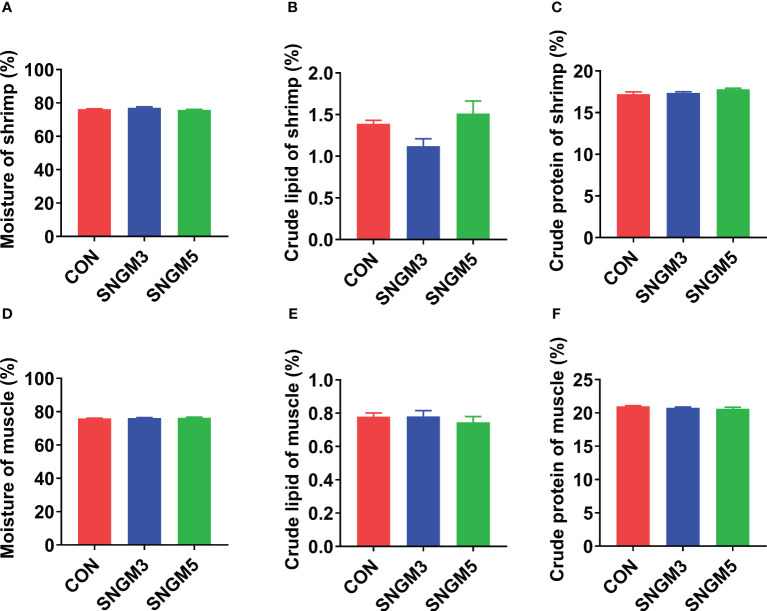
Effects of SNGM on the proximate compositions of the whole shrimp and muscle. **(A)** Moisture content of the whole shrimp. **(B)** Crude lipid content of whole shrimp. **(C)** Crude protein content of the whole shrimp. **(D)** Moisture content of the muscle. **(E)** Crude lipid content of the muscle. **(F)** Crude protein content of the muscle. Values are means ± SEM.

As shown in **Figure 2**, the proximate compositions of whole shrimp and muscle were measured. There were no significant effects on moisture, crude lipid, and crude protein contents of whole shrimp and muscle between the CON and SNGM groups (*P* > 0.05).

### 3.3 The immune parameters of hemolymph

**Figure 3:**
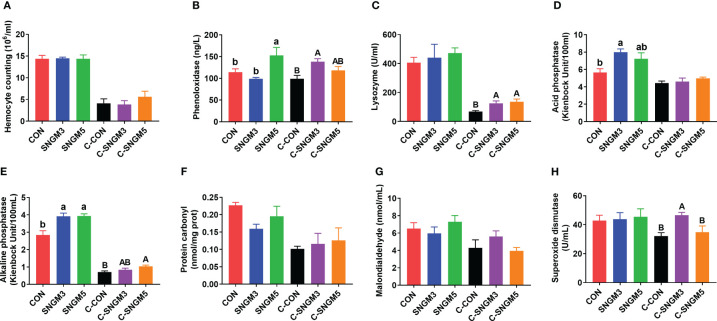
Effects of SNGM on the immune parameters of the hemolymph. **(A)** Hemocyte count. **(B)** Phenol oxidase activity. **(C)** Lysozyme activity. **(D)** Acid phosphatase activity. **(E)** Alkaline phosphatase activity. **(F)** Protein carbonyl content. **(G)** Malonaldehyde content. **(H)** Superoxide dismutase activity. Growth experiment groups: CON, SNGM3, and SNGM5. Challenge experiment groups: C-CON, C-SNGM3, and C-SNGM5. Values are means ± SEM. In the growth experiment, values with different lowercase letters are significantly different (*P* < 0.05). In the challenge experiment, values with different capital letters are significantly different (*P* < 0.05).

At the end of the growth trial, compared to the CON group, the acid phosphatase and alkaline phosphatase activities were significantly increased in the SNGM3 group (**Figures 3D, E**). The phenol oxidase and alkaline phosphatase activities were significantly increased in the SNGM5 group (*P* < 0.05) (**Figures 3B, E**). There were no significant differences in hemocyte count, protein carbonyl, and malonaldehyde contents as well as lysozyme and superoxide dismutase activities between the CON and SNGM groups (*P* > 0.05) (**Figures 3A, C, F, G, H**).

In the challenge experiment, the phenol oxidase, lysozyme, alkaline phosphatase, and superoxide dismutase activities in the SNGM groups were higher than in the CON group (*P* < 0.05) (**Figures 3B, C, E, H**), and SNGM did not affect the hemocyte count, acid phosphatase activity, protein carbonyl content, and malonaldehyde content (*P* > 0.05) (**Figures 3A, D, F, G**). Compared with normal-growth groups, *Vibrio* challenge reduced the hemocyte count, and activities of lysozyme, acid phosphatase, and alkaline phosphatase (**Figures 3A, C-E**).

### 3.4 The immune parameters and health status of the hepatopancreas

**Figure 4:**
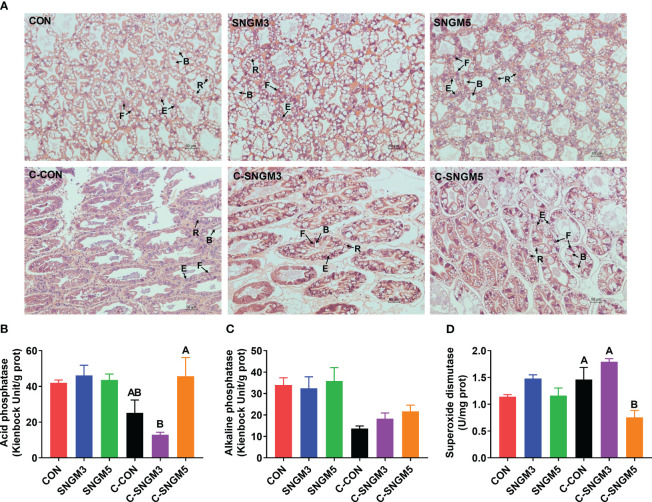
Effects of SNGM on the histology and immune parameters of the hepatopancreas. **(A)** Histological analysis (H&E staining) of the hepatopancreas. Black arrows mark the four cell types of tubules, including B (“blasenzellen”) cells that typically contain a single, large secretory vesicle, R (“restzellen”) cells that usually contain variable-sized lipid vacuoles, F (“fibrillenzellen” or fibrous) cells that contain a large number of ribosomes and a well-developed endoplasmic reticulum, and E (“embryonalzellen” or embryonic) cells that are the main cell types at the tip of the distal hepatopancreatic tubule. **(B)** Acid phosphatase activity. **(C)** Alkaline phosphatase activity. **(D)** Superoxide dismutase activity. Growth experiment groups: CON, SNGM3, and SNGM5. Challenge experiment groups: C-CON, C-SNGM3, and C-SNGM5. Values are means ± SEM. In the challenge experiment, values with different capital letters are significantly different (*P* < 0.05).

At the end of the growth trial, the hepatopancreas cells in all groups are star-shaped and regularly arranged, but there was an increase in histological status in group SNGM5 compared to the control (**Figure 4A**). After the challenge experiment, the hepatic corpuscles deformed to some extent, the tubule epithelial cells fell off, and the lumen was irregular and atrophied, but the SNGM3 and SNGM5 groups revealed better conditions (**Figure 4A**). At the growth trial, there were no significant differences in the activity of acid phosphatase, alkaline phosphatase, and superoxide dismutase, respectively, between the CON and SNGM groups (*P* > 0.05) (**Figures 4B–D**). In the challenge experiment, the SNGM5 group had a higher acid phosphatase activity and a lower superoxide dismutase activity than the CON group (*P* < 0.05) (**Figures 4B, D**).

### 3.5 Nutrient levels of the hemolymph and hepatopancreas

**Figure 5:**
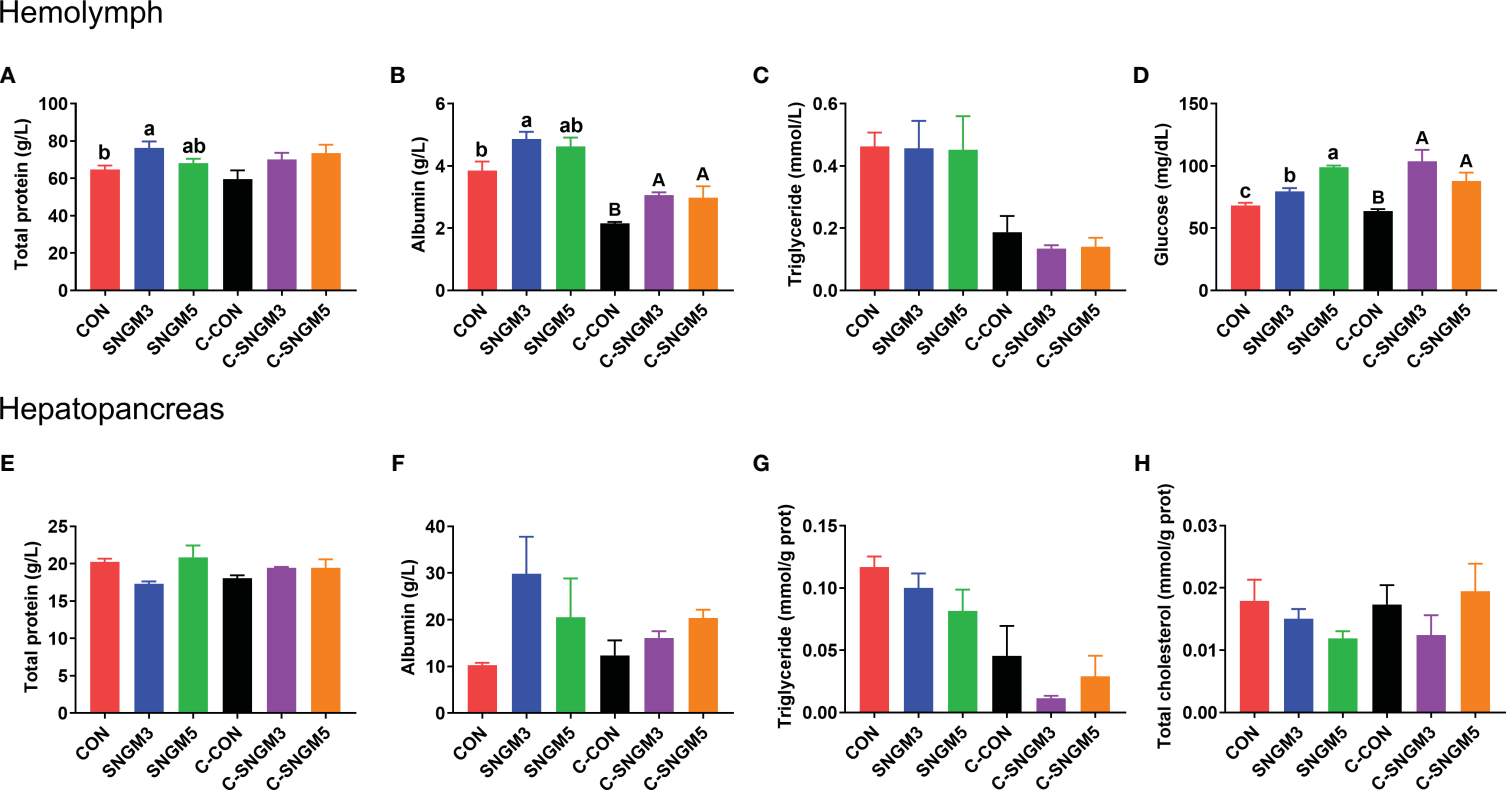
Effects of SNGM on nutrient metabolism of the hemolymph and hepatopancreas. **(A)** Total soluble protein content in the hemolymph. **(B)** Albumin content in the hemolymph. **(C)** Triglyceride content in the hemolymph. **(D)** Glucose content in the hemolymph. **(E)** Total soluble protein content in the hepatopancreas. **(F)** Albumin content in the hepatopancreas. **(G)** Triglyceride content in the hepatopancreas. **(H)** Total cholesterol content in the hepatopancreas. Growth experiment groups: CON, SNGM3, and SNGM5. Challenge experiment groups: C-CON, C-SNGM3, and C-SNGM5. Values are means ± SEM. In the growth experiment, values with different lowercase letters are significantly different (*P* < 0.05). In the challenge experiment, values with different capital letters are significantly different (*P* < 0.05).

At the growth trial, compared with the CON group, the total soluble protein and albumin concentrations in the hemolymph were significantly increased in the SNGM3 group (*P* < 0.05). The SNGM5 group also resulted in an increase in numerical values, but no significant difference was observed (*P* > 0.05) (**Figures 5A, B**). Compared with the CON group, dietary SNGM significantly increased the hemolymph glucose content in both the growth trial and the challenge experiments (*P* < 0.05) (**Figure 5D**). In the challenge experiment, compared with the CON group, the SNGM supplementation increased the total soluble protein and albumin concentrations in both hemolymph and hepatopancreas (**Figures 5A, E, F**), and a significant difference was found in the hemolymph albumin concentration (*P* < 0.05) (**Figure 5B**). In addition, the SNGM supplementation did not affect the triglyceride content in the hemolymph and hepatopancreas as well as the total cholesterol content in the hepatopancreas (*P* > 0.05) (**Figures 5C, G, H**).

### 3.6 The immune parameters and morphology of the intestine

**Figure 6:**
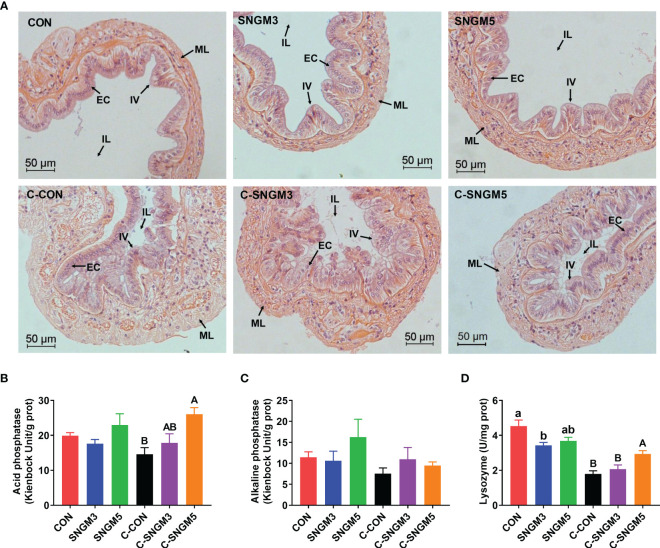
Effects of SNGM on the histology and immune parameters of the intestine. **(A)** Histological analysis (H&E staining) of the intestine. Black arrows mark the intestinal villi (IV), epithelial cell (EC), intestinal lumen (IL), and muscle layer (ML) of the intestine. **(B)** Acid phosphatase activity. **(C)** Alkaline phosphatase activity. **(D)** Lysozyme activity. Growth experiment groups: CON, SNGM3, and SNGM5. Challenge experiment groups: C-CON, C-SNGM3, and C-SNGM5. Values are means ± SEM. In the growth experiment, values with different lowercase letters are significantly different (*P* < 0.05). In the challenge experiment, values with different capital letters are significantly different (*P* < 0.05).

The representative slices of middle intestine histology are shown below. In the growth trial, there were no significant differences in the intestinal morphology among the CON, SNGM3, and SNGM5 groups (*P* > 0.05) (**Figure 6A**). However, after the challenge experiment, the intestinal folds and epithelial cells of the CON group were damaged, atrophied, and exfoliated, but the SNGM alleviated the adverse effects induced by *Vibrio parahaemolyticus.* In the growth trial, compared to the CON group, the SNGM supplementation reduced the lysozyme activity in the intestine, and significance was found between CON and SNGM3 (*P* < 0.05) (**Figure 6D**). In the challenge experiment, SNGM5 significantly improved the acid phosphatase and lysozyme activities in the intestine (*P* < 0.05) (**Figures 6B, D**). Besides, the SNGM supplementation did not affect the alkaline phosphatase activity in the intestine (**Figure 6C**).

### 3.7 The composition of intestinal microbiota

**Figure 7:**
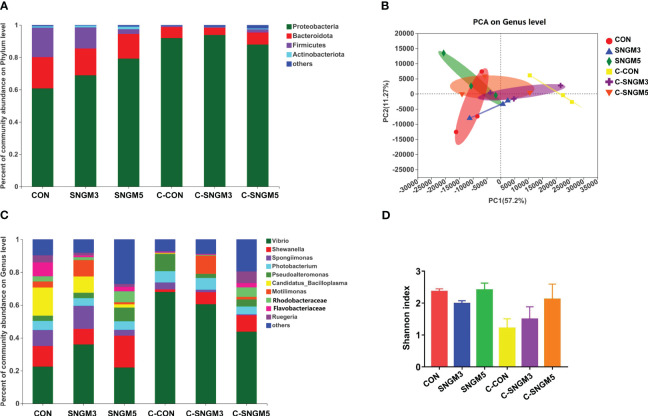
Effects of SNGM on intestinal microbiota. **(A)** Percent of community abundance on the phylum level. **(B)** Principal component analysis (PCA) on the genus level. **(C)** Percent of community abundance on genus the level. **(D)** Shannon index on the genus level. Growth experiment groups: CON, SNGM3, and SNGM5. Challenge experiment groups: C-CON, C-SNGM3, and C-SNGM5.

The percent of community abundance on the phylum level (**Figure 7A**) showed that Proteobacteria, Bacteroidota, Firmicutes, and Actinobacteria were dominant in the gut of shrimps. At the growth trial, compared with the CON group, the abundance of Proteobacteria and Actinobacteria increased while those of Bacteroidota and Firmicutes decreased in the SNGM3 and SNGM5 groups. Compared with the growth groups, after the challenge experiment, the abundance of Proteobacteria increased, but those of Bacteroidota, Firmicutes, and Actinobacteria all decreased (**Figure 7A**). There was no significant difference in these parameters among the control, SNGM3, and SNGM5 groups in both the growth trial and the challenge experiments (*P* > 0.05). The PCA analysis suggested that the composition of intestinal microbiota was similar among the CON, SNGM3, and SNGM5 groups in the growth trial, but it was obviously different after the challenge experiment, especially between the C-CON and C-SNGM5 groups (**Figure 7B**). Compared to C-CON, the C-SNGM5 group showed a shorter distance to the normal growth trial groups, which indicates generally normal intestinal microbiota.

The dominant bacteria genus abundance (top 10) is shown in **Figure 7C**; **Table 2**. At the growth trial, there was no difference in the abundance of the 10 bacteria between the CON group and SNGM groups (*P* > 0.05). Compared with the growth trial, the *Vibrio* abundance was increased in all challenge experiment groups. After the challenge experiment, the abundance of *Vibrio*, *Photobacteriuma*, *Pseudoalteromonas*, and *Candidatus_Bacilloplasma*, which belong to pathogenic bacteria, in the C-SNGM3 and C-SNGM5 groups, respectively, was lower compared to the C-CON group, while the abundance of *Shewanella*, which belongs to probiotic bacteria, was increased by SNGM. Compared with the growth trial, the community diversity (Shannon index) in the challenge experiment decreased slightly. In the challenge experiment, the community diversity of SNGM groups was slightly higher compared to the CON group (**Figure 7D**).

**Table 2:** Percent of community abundance on genus level (top 10).

Genus level	CON	SNGM3	SNGM5	C-CON	C-SNGM3	C-SNGM5
*Vibrio*	22.56 ± 4.45	36.88 ± 8.08	26.42 ± 14.21	68.18 ± 8.56	58.42 ± 10.87	44.16 ± 18.19
*Shewanella*	12.31 ± 3.82	9.25 ± 2.41	17.91 ± 10.68	1.55 ± 0.89	6.86 ± 3.15	9.74 ± 6.59
*Photobacterium*	5.77 ± 2.30	4.35 ± 2.75	5.34 ± 0.98	6.53 ± 5.76	8.81 ± 8.62	4.66 ± 3.44
*Spongiimonas*	10.15 ± 4.15	12.98 ± 9.24	3.67 ± 0.73	4.37 ± 2.45	1.57 ± 0.43	0.54 ± 0.28
*Pseudoalteromonas*	3.02 ± 1.90	3.40 ± 0.82	8.11 ± 5.54	10.5 ± 7.65	2.71 ± 1.16	4.17 ± 2.45
Candidatus_Bacilloplasma	17.4 ± 8.87	9.84 ± 0.39	2.33 ± 0.99	0.29 ± 0.26	0.01 ± 0.01	NA
*Motilimonas*	3.58 ± 1.61	10.58 ± 7.89	1.14 ± 0.58	0.24 ± 0.10	10.91 ± 10.64	1.62 ± 1.1
*Rhodobacteraceae*	3.00 ± 0.76	1.65 ± 0.85	5.54 ± 4.17	0.19 ± 0.16	0.45 ± 0.26	5.78 ± 5.28
*Flavobacteriaceae*	8.02 ± 6.16	1.20 ± 0.56	2.37 ± 1.32	0.74 ± 0.42	0.52 ± 0.32	2.57 ± 1.46
*Ruegeria*	4.47 ± 1.51	1.69 ± 0.42	1.56 ± 0.73	0.05 ± 0.03	0.28 ± 0.18	7.27 ± 7.23

Feeding experiment groups: CON, SNGM3, and SNGM5. Challenge experiment groups: C-CON, C-SNGM3, and C-SNGM5. Data are mean ± SEM (n = 3).

## 4 Discussion

In recent years, with the development of intensive aquaculture, aquatic animals are more vulnerable to environmental stresses and pathogen infection, which impaired the animal growth, metabolism, antioxidation capacity, and immunity (2, 23, 24). The AHPND outbreak took place firstly in China in 2009 and then spread to Vietnam, Malaysia, Thailand, and Mexico, which caused enormous economic losses (25). Health-promoting additives have been used in animal feeds for decades as antimicrobial and growth promoter. However, few information was available about their combination use in the shrimp farming industry. In the present study, we evaluated the use of SNGM, a commercially available functional feed additive, in Pacific white shrimp diets. Our results showed that dietary SNGM, especially at 3 g/kg feed, promoted growth and resistance to *Vibrio*. After challenge by *Vibrio*, the SNGM also resulted in higher activities of phenol oxidase, lysozyme, alkaline phosphatase, and superoxide dismutase than the CON group. These data all clearly demonstrated that the phytobiotic-based compound additive (Sanacore® GM) could improve the growth, immunity, antioxidant capacity, and resistance to pathogenic bacteria in Pacific white shrimp. Similar results were observed in other previous studies. Plant extracts have been widely used in aquaculture to reduce the dependence on antibiotics and promote the health status of shrimp (12, 26, 27). Pacific white shrimp immersed in the seawater containing 400 or 600 mg/L plant (*Gracilaria tenuistipitata*) extract for 3 h had significantly improved levels of hemocyte count, respiratory burst, lysozyme, phenoloxidase, and superoxide dismutase as well as higher survival after WSSV challenge (28). In black tiger shrimp (*Penaeus monodon*), compared with control diet, feeding diets with methanolic extracts of nine plants at 2.5 mL/kg for 60 days resulted in higher survival during the growth process, specific growth rate, feed efficiency, and survival after *Vibrio harveyi* infection (29). In Pacific white shrimp, oral (10 g/kg feed for 14 days) or intramuscular (50 ug/g body weight for 48 h) administration of galangal extract all significantly increased the relative expression of immune-related genes in the hemocytes and survival after *Vibrio harveyi* infection (30). Organic acids also play beneficial roles by inhibiting intestinal pathogens’ growth. Sodium acetate, sodium butyrate, and sodium propionate could inhibit the growth of three *Vibrio* species (*Vibrio harveyi*, *V. alginolyticus*, and *V. anguillarum*) *in vitro* (TCBS agar). Pacific white shrimp diets containing 2% sodium propionate reduced the *Vibrio* species concentration in the intestinal microbiota and increased the feed palatability and apparent digestibility of dry matter and phosphorus (31). Yeast extract is also recognized as immune-stimulant. In juvenile Pacific white shrimp, dietary yeast extract supplementation at 2.0% increased the weight gain, specific growth rate, condition factor, total antioxidant capacity, catalase and glutathione peroxidase activities, and microbiota diversity (Shannon indexes) (32). Yeast extract could replace 45% of the fish meal in Pacific white shrimp diet (containing 25% fish meal) and did not affect weight gain and muscle composition (33). In particular, our results proved that the combination of functional ingredients improved the growth and immunity in Pacific white shrimp.

Disease outbreak is a major threat to the sustainable development of shrimp farming. At present, among shrimp pathogens, *Vibrio parahaemolyticus* is the most severe one, which could result in AHPND. This disease, which causes a pale and atrophied hepatopancreas with an empty gastrointestinal tract in shrimp and further causes mortality, has led to a large economic loss in white shrimp farming (2, 34). In the study, SNGM showed promising performance in resistance to *Vibrio parahaemolyticus* infection. In Pacific white shrimp, the histological analysis of AHPND showed that the tubule epithelial cells of hepatopancreas fell off into the tubule lumens (22). Similar results were also found in the challenge experiment of the present study. After challenge by *Vibrio parahaemolyticus*, the hepatic corpuscles deformed to some extent, the tubule epithelial cells fell off, and the lumen was irregular and atrophied, but the use of SNGM alleviated the adverse conditions and increased the total soluble protein and albumin concentrations in the hepatopancreas. This was similar to the previous studies demonstrating the relevant efficacy of individual use of health additives in shrimp and, more broadly, fish (35, 36). These results suggest that SNGM could improve shrimp health by maintaining the morphological structure of both hepatopancreas and intestine. In yellow drum (*Nibea albiflora*), dietary *Bacillus subtilis* B0E9 and *Enterococcus faecalis* AT5 increased the resistance to red-head disease challenged by *Vibrio harveyi* B0003 by improving the liver morphology, serum and skin immunity, and intestine and skin mucosal microbiota composition (37). There have been few studies investigating the relationship between health additives and gut microbiota after *Vibrio* challenge in shrimp. We found that Proteobacteria, Bacteroidota, Firmicutes, and Actinobacteria were the dominant phyla in the intestine of Pacific white shrimp. In the growth trial, the SNGM addition increased the abundance of Proteobacteria and Actinobacteria but decreased the abundance of Bacteroidota and Firmicutes. The *Vibrio parahaemolyticus* challenge increased the Proteobacteria abundance but reduced the abundance of Bacteroidota, Firmicutes, and Actinobacteria. Meanwhile, the community diversity (Shannon index) was decreased slightly after challenge. A study on Chinese mitten crab (*Eriocheir sinensis*) showed that the hepatopancreas necrosis disease also reduced the abundance of phyla Firmicutes (38). Regulation of intestinal microbiota by plant extracts or organic acids has been reported in limited studies. The addition of bamboo leaf flavonoids (500 and 1,000 mg/kg) in crab diets decreased the Shannon (diversity) and Chao (richness) indexes as well as the phyla Bacteroidota abundance (39). A study on Pacific white shrimp showed that diets supplemented with a mixture of organic acids increased the microbiota diversity and richness in the gut and the abundance of Firmicutes and *Lactobacillus* but reduced the abundance of Proteobacteria (40). In shrimp primary cells, a mixture of organic acids increased the probiotic *Faecalibacterium prausnitzii* content, short-chain fatty acid level in the gut, and resistance to *V. parahaemolyticus* (41).

In the present study, compared with the growth trial, the *Vibrio* abundance was increased in all challenge experiment groups. After the challenge experiment, the SNGM groups had lower *Vibrio*, *Photobacteriuma*, *Pseudoalteromonas*, and *Candidatus_Bacilloplasma* abundance in the gut than the C-CON group, while these had higher *Shewanella* abundance and community diversity than the C-CON group. The PCA analysis also showed that the intestinal microbiota of the C-SNGM5 group showed a shorter distance to the normal growth trial groups than the C-CON group. Previous studies have shown that *Vibrio*, *Photobacteriuma*, *Pseudoalteromonas*, and *Candidatus_Bacilloplasma* are pathogenic bacterium and usually accumulate in the gut of diseased shrimp, but the *Shewanella*, *Chitinibacter*, and *Rhodobacter* abundance is higher in healthy shrimp (42). In shrimp with white feces syndrome (WFS), the intestinal *Paracoccus* and *Lactococcus* abundance and the bacterial diversity were reduced, whereas the abundance of *Candidatus, Bacilloplasma*, and *Phascolarctobacterium* was increased (43). Dietary sulfate-based alginate polysaccharide (2% to 3% for 56 days) improved the intestinal health and *Vibrio parahaemolyticus* resistance by decreasing the *Vibrio*, *Pseudoalteromonas*, and *Candidatus Bacilloplasma* abundance in Pacific white shrimp fed low-fishmeal diets (44). All of these results clearly indicate that the use of SNGM could promote intestinal health by increasing the probiotics level and reducing the level of harmful bacteria.

## 5 Conclusion

Dietary phytobiotic-based additive at 3-g/kg level significantly increased the growth and survival of Pacific white shrimp after *Vibrio parahaemolyticus* challenge. The immune-related enzyme activities, morphological structure of hepatopancreas and intestine, hepatopancreas metabolism and intestinal microbiota composition were also improved by adding 3–5 g/kg of additive. The results suggest that the suitable dosage of the phytobiotic-based additive (Sanacore® GM) is 3-g/kg feed to promote shrimp growth and disease resistance in practical applications.

## Data Availability

The data presented in the study are deposited in the NCBI repository, accession number PRJNA1055185. Further inquiries can be directed to the corresponding author.
